# Paramedics’ knowledge, attitudes, and practices regarding the use of personal protective equipment against COVID-19

**DOI:** 10.5339/qmj.2022.50

**Published:** 2022-11-03

**Authors:** Padarath Gangaram, Yugan Pillay, Guillaume Alinier

**Affiliations:** ^1^Ambulance Service, Hamad Medical Corporation, Doha, Qatar. Email & ORCID ID: pgangaram@hamad.qa & https://orcid.org/0000-0001-5282-5045H; ^2^Honorary Research Fellow, Faculty of Health Sciences, Durban University of Technology, South Africa; ^3^University of Hertfordshire, Hatfield, UK

**Keywords:** COVID-19 knowledge, COVID-19 transmission, novel coronavirus disease, paramedics’ attitudes, personal protective equipment practice

## Abstract

The risk of novel coronavirus disease (COVID-19) transmission in the confined mobile ambulance compartment is increased during aerosol-generating procedures and close proximity. Paramedics are encouraged to increase body-surface-isolation by donning additional personal protective equipment (PPE) during patient encounters. This study aimed to better understand paramedics’ knowledge, attitudes, and practices related to PPE use during the COVID-19 pandemic in the prehospital setting with a focus on mitigating risks associated with infection control.

This prospective quantitative study collected descriptive data using a specifically designed data collection tool. The survey data was then cleaned and analyzed with Microsoft Excel® and the latest version of the Statistical Package for Social Sciences.

One thousand frontline paramedics employed by the Hamad Medical Corporation Ambulance Service (HMCAS) were invited via email to participate in the study. A total of 282 (28.2% of frontline paramedics) paramedics completed the online survey, of which 80.1% completed the mandatory HMCAS online infection control training program within the last year, and 17.0% between one to two years ago. Approximately 83% of the participants had completed an N95 mask fit test at HMCAS within the past five years, and 91.5% completed the hand hygiene training.

The study found that 98.2% of the paramedics were knowledgeable about COVID-19 and its transmission, while 96.1% agreed that aerosol-generating procedures increased airborne transmission. The paramedics’ attitudes were mainly positive toward the use of PPE to prevent the spread of the virus, which was synchronous with their practice. The sample population demonstrated a strong knowledge of COVID-19 and its transmission. Their overall positive attitudes and good infection control practices were demonstrative of efforts to mitigate risks associated with the spread of the virus.

## Introduction

Since the declaration as a pandemic of the disease caused by the severe acute respiratory syndrome coronavirus-2 (SARS-CoV-2) virus, its spread has been presenting at different phases and variants in various countries and territories worldwide. The common denominator internationally to date has been the incredible demand on healthcare personnel and resources, with increased emphasis on the use of personal protective equipment (PPE) by paramedics to mitigate transmission risks. At the Hamad Medical Corporation Ambulance Service (HMCAS), no study has thus far been conducted to determine paramedics’ knowledge, attitudes, and practices related to PPE use against COVID-19, other than a study looking broadly at staff readiness to respond to hazardous material chemical, biological, radiological, and nuclear (HazMat-CBRN) incidents.^1^

Public emergency medical services in the State of Qatar are provided by the HMCAS. The national operator utilizes the hub and spoke model to ensure that the public receive rapid access to effective emergency care.^2^ The two categories of staff employed by the HMCAS include Critical Care Paramedics (CCPs) and Ambulance Paramedics (APs). CCPs are commonly trained to provide the highest level of care in a prehospital environment and are knowledgeable and skillful in performing various invasive and non-invasive procedures, as highlighted in the Clinical Practice Guidelines.^3^ APs are primarily recruited from Tunisia, India, Philippines, Jordan, Morocco, Egypt, and the United Kingdom. Within the State of Qatar, the CCPs and APs function in numerous roles, including clinical operations, aeromedical, management, education, and specialized medical services.^4^

All new recruits employed at the HMCAS are mandated to complete a four-hour online infection control training program, which includes effective donning and doffing of PPE. They are subsequently required to complete the online infection control program annually. New recruits undergo a mandatory N95 mask fit test upon employment, to determine their correct N95 mask size. This consists of paramedics using their sense of smell or taste while a tester vaporizes a test solution under a hood paramedics wear during the procedure, to determine which size of mask best fits their face and avoid any leakage. As part of the onboarding program, the staff also undergo a two-hour hand hygiene training, at the end of which they are required to correctly demonstrate the appropriate procedures for hand hygiene.

It has been more than two years since COVID-19 was first reported to the World Health Organization (WHO).^5^ The data as of March 23, 2022 show that more than 470 million persons have been infected with COVID-19 in more than 225 countries and territories. More than six million people have died globally after testing positive for COVID-19, and there are approximately 1.5 million new cases daily.^6^

The number of healthcare workers (HCWs) infected with COVID-19 has increased^7^ and many lost their lives due to the disease.^8^ The reasons are multifactorial.^9^ The primary concerns are related to delays in recognition of cases, lack of PPE, and improper donning and doffing of PPE.^10^

Currently, there are seven major strains of coronavirus, but only three may produce symptoms that can be life-threatening—Middle East respiratory syndrome-related coronavirus (MERS-CoV), severe acute respiratory syndrome coronavirus (SARS-CoV), and the latest, SARS-CoV-2.^5^ SARS-CoV-2 has been shown to spread between people who are in close contact with each other.^5,11^ Small droplets produced when an infected person coughs, sneezes, talks, or exhales fall to the ground or onto surfaces.^5,6^ People may inhale the droplets or touch the contaminated surfaces and then touch their eyes, nose or mouth, inadvertently contributing to the spread of the virus,^12^ which is most contagious in the first three days after the presentation of symptoms.^12^

Paramedics are at a great risk of exposure to SARS-CoV-2, which causes the coronavirus disease.^11^ Paramedics routinely perform life-saving treatment in close proximity, and aerosol-generating procedures on critically ill/injured patients in an emergency environment.^11,13^ Procedures such as non-invasive ventilation (NIV), endotracheal intubation (ETI), and external chest compressions (ECC) result in aerosol generation, and, in a confined ambulance compartment, has an increased risk of virus transmission^11^ due to virus particles being propelled into the air.^6,8,14^ Particles smaller than five microns remain suspended in the air for protracted periods.^6,11^ Paramedics are therefore encouraged to don additional PPE to increase body-surface-isolation—high-filtration face masks, face shields, surgical gowns, double gloves, and surgical hoods—during encounters with patients with suspected or confirmed SARS-CoV-2 infection.^6,11,15^

## Study Objective

This study aimed to better understand paramedics’ knowledge, attitudes, and practices related to PPE use during the COVID-19 pandemic in the prehospital setting, with a focus on mitigating risks associated with infection control.

## Methods

### Infection control measures at HMCAS

Paramedics, as first responders at the HMCAS in Qatar, are at the forefront of the COVID-19 pandemic, including being involved in the testing and vaccination processes.^16^ In the prehospital setting, paramedics attend to the sick and injured presenting with various emergencies in the community or during inter-facility transfers.^17^ Patients presenting with general clinical emergencies are now approached as suspected COVID-19 cases, prompting paramedics to don additional PPE.^18^

The HMCAS previously strategized to mitigate risks associated with Ebola and MERS-CoV. These programs were afoot to reduce infection risks and protect staff prior to the advent of the COVID-19 pandemic. Current infection control practices for COVID-19 mandate that HMCAS vehicles and emergency medical equipment undergo special cleaning with 70% alcohol wipes at the start of every shift and after completion of each emergency service call. All ambulances that transport a suspected or confirmed COVID-19 patient are removed from service and undergo deep cleaning by the HMCAS Support Services section.

### Personal protective equipment

HMCAS paramedics are required to don double gloves, an N95 mask, a surgical gown, face shield, and surgical hood when managing and transporting all suspected or confirmed COVID-19 patients. Upon completion of the emergency call, the paramedics are required to doff the PPE in a safe manner, according to stringent HMCAS standard operating procedures.

Donning of PPE by paramedics protects other healthcare workers that the paramedic comes into contact with during their duty,^19^ and provides protection to patients and the greater community from a possible contagious paramedic.^15^ Finally, the paramedic that appropriately dons the required PPE against COVID-19 protects themselves from contracting the virus. Failure to don the appropriate PPE correctly places the paramedic and all their social contacts at risk of contracting COVID-19.^15,20^

This prospective quantitative study focused on collecting descriptive data, utilizing a purpose-designed online survey sent to HMCAS frontline clinicians. Once Medical Research Centre approval to conduct the research was received, the online questionnaire link was distributed—in July 2020. Completion of the online survey was voluntary, and the responses were anonymized. The study was conducted when the country had experienced the first wave of COVID-19 infections. All Department of Healthcare Professions licensed frontline APs and CCPs employed by the HMCAS were invited to participate in the study (target population n =  1,000). Slovin's formula was used to calculate the sample size. We determined that we needed to recruit 282 participants at a 95% confidence level.

A specific data collection tool was designed by the researchers for this study since there was no other known tool found to be fit-for-purpose within the novel coronavirus context, and further embedded in a prehospital context. The survey tool was assessed for content and face validity by an expert panel within the HMCAS, comprised of experienced APs and CCPs with a special focus on infection control and medical response in hazardous materials environments. The survey was hosted on the Google® Documents online platform. Identified staff was emailed invitations to participate in the study, with an access link to the survey tool. The study participants were reminded to complete the online survey at 7 and 14 days after the initial email invitation. The staff was also encouraged to only complete the survey once and then ignore the reminder emails.

Microsoft Excel® and the 26^th^ version of the Statistical Package for Social Sciences (SPSS^®^) were used to clean and analyze the survey data. All non-HMCAS and non-DHP licensed staff were excluded from the study.

## Results

As per the sample size calculation requirement, data collection was ended once 282 paramedics had completed the online survey. Of this sample, 90.4% (n = 255) were male, 78.7% (n = 222) had a bachelor's degree, and 9.9% (n = 28) had a diploma or master's degree. Most of the participants were APs (92.2%, n = 260); 35.8% (n = 101) had been employed at the HMCAS for 6 to 10 years, 34.8% (n = 98) for 1 to 5 years, and 12.8% (n = 36) for less than one year.

Approximately 80.1% (n = 226) completed the mandatory HMCAS online infection control training program less than one year ago, and 17.0% (n = 48) between one to two years ago. Meanwhile, 83% completed an N95 mask fit test at the HMCAS within the last five years—30.1% within less than one year, 29.4% from one to two years, and 23.4% from three to five years. Similarly, 91.5% had completed the hand hygiene training program within the last five years—50.7% within less than one year, 25.9% from one to two years, and 14.9% from three to five years.

The paramedics’ knowledge on COVID-19 and use of PPE is summarized (Table 1). The paramedics agreed that procedures such as NIV (90.8%), ECC (85.8%), and High Flow Nasal Cannula (HFNC) (70.6%) increased the risk of aerosolization of COVID-19 particles, and 96.1% agreed that aerosol-generating procedures increased airborne transmission.

The participants’ attitudes—positive or negative—toward the use of PPE for COVID-19 patients in the prehospital environment was evaluated (Table 2). Ventilation using a bag valve mask (BVM) was identified as an aerosol-generating procedure. Of the participants, 92.9% agreed that a heat and moisture exchanger (HME) filter is required during BVM ventilation. Further, to halt transmission of the virus, 95.4% agreed that the practice of hand hygiene is required after doffing PPE to protect from the transmission of COVID-19. Only 61.3% agreed that donning of PPE when managing or transporting a suspected or confirmed COVID-19 patient should be done under supervision, while 47.1% agreed that the doffing of PPE should be done on-scene before transporting a suspected or confirmed COVID-19 patient.

Paramedic practices when treating suspected or confirmed COVID-19 patients were evaluated based on their responses to PPE use in the prehospital environment (Table 3). These responses were self-reported by the paramedics—99.6% reported that they don PPE before contacting a suspected or confirmed COVID-19 patient; 91.5% reported that they do not remove their N95 mask when performing manual ECC, although the procedure is exhausting. Meanwhile, 80.1% reported that they follow HMCAS standard operating procedures (SOPs) by having their supervisor witness and check the doffing of PPE to ensure they are following the appropriate doffing technique.

### Discussion

In this study, the paramedics demonstrated that they had good knowledge of COVID-19 and its spread. Further, they showed a positive attitude toward the appropriate donning and doffing of PPE and mitigating the risk of spread of the virus.

The risks associated with the spread of COVID-19 among paramedics is high due to their exposure and performance of aerosol-generating procedures^21^ when managing suspected or confirmed COVID-19 patients within the confined ambulance compartment.^11^ Therefore, paramedics’ knowledge, attitudes, and practices related to proper PPE use against COVID-19 in the prehospital environment is important in mitigating the risks associated with virus transmission.^20,22^ As first responders, paramedics are the initial practitioners who identify suspected or confirmed COVID-19 patients, informing the risks the patient may pose and appropriate PPE use by the entire healthcare chain.^17,23^

One hundred percent of the participants in the study held at least a diploma qualification in a health-related field. Having probably adhered to the institutional training program requirements, these paramedics’ prior knowledge on infection control measures was reflected in their strong knowledge of the virus and its transmission. Other related studies revealed similar results on healthcare workers’ knowledge of PPE use in the context of the COVID-19 pandemic.^10,21,24^ Regular completion of the organization-mandated programs further embedded the culture of infection control. Although these programs were compulsory, no such program, among the participants in this study, achieved 100% compliance. This may be attributed to the pandemic, increased operational demand for resources, and the disruption of educational programs within the organization.

The paramedics’ knowledge of the virus and its spread informed their attitudes toward the proper use of PPE for suspected or confirmed COVID-19 patient encounters. The paramedics’ positive attitudes were demonstrated by their need to perform proper hand hygiene, double-gloving, and wearing a face shield to prevent the spread of the virus. However, only approximately two-thirds (61.3%) of the paramedics agreed that the donning of PPE should be done under supervision, demonstrating their partially negative attitude to this practice. The HMCAS SOP advocates the donning and doffing of PPE for infectious diseases under supervision, to comply with the appropriate procedure and reduce the risk of further infection and virus transmission. During the first wave of the pandemic in the State of Qatar, prehospital resources were in extreme demand. Emergency call volumes doubled; thus, the availability of an on-scene supervisor was not always possible. The HMCAS SOP was written with the care and transport of infectious disease patients in mind. The pandemic, however, posed different challenges with the increased resource demand. Staff was encouraged to observe each other properly donning PPE prior to patient contact instead of relying on an actual supervisor.

The paramedics’ negative attitude was recorded when less than 50% (47.1%) of the paramedics indicated that PPE should be doffed on-scene before transporting a suspected or confirmed COVID-19 patient. This response could be attributed to the two-person paramedic team, whereby the first AP accompanies the patient in the patient compartment during transport to hospital, while the second AP drives the ambulance. The inappropriate doffing of PPE increases the paramedic's risk of exposure and further spread of the virus during the patient transport phase.^23^ After treating and transporting an infectious disease patient, PPE should be doffed in a safe zone.^11^ Doffing of PPE at the HMCAS was observed by a supervisor in a safe zone at the hospital or at specific disinfection sites.

Good paramedic practices when treating suspected or confirmed COVID-19 patients were gauged based on their response to PPE use in the prehospital environment. In light of the pandemic, the moral practice of always donning proper PPE prior to all patient contact is essential to prevent the spread of the virus.^18,20^ Maintaining full PPE use during the entire treatment and transportation phase in the prehospital environment is critical.^23^ A large proportion (83%) of the paramedics reported that in their practice, they should not remove their N95 mask when performing manual ECC, although the procedure is exhausting. The average temperature in Qatar in June 2020 was 41.2 degrees Celsius. The extreme weather poses many prehospital operational challenges for ambulance services.^25,26^ Paramedics endure the inclement weather and have to don full PPE when performing manual ECC.^26^ They were advised to rotate manual ECC between staff after each two-minute cycle to maintain its effective performance.

The paramedics self-reported good practices when they reported that the removal of N95 masks should not be performed in the back of the ambulance when treating or transporting a suspected or confirmed COVID-19 patient. The use of a face shield for the aerosol-generating ETI procedure is essential;^6,23^ 77.3% disagreed with removing their face shield during the procedure.

An indirect possible effect of this survey is that it made participants reflect on their clinical practice in the context of the pandemic. However, we are unsure if it will positively or negatively influence their future use of PPE. Regular HMCAS staff directives have been published and disseminated to remind all staff of expected PPE use in various circumstances. The HMCAS also ensured that infection control training programs were tailored and regularly updated according to recommended practices in the context of COVID-19. A follow-up action we should have rapidly considered is to display the survey findings across our network of ambulance hubs and spoke stations, clearly highlighting the expected correct answers post survey completion. The findings of this study will also help in the formulation of educational materials, should we encounter a similar medical crisis in the future.

## Conclusion

An effective model for curbing the spread of COVID-19 must consider the knowledge, attitudes, and practices of paramedics as first responders. During a pandemic when so many individuals may be asymptomatic, the safest measure is for clinicians to take all possible precautionary measures, as they can themselves become a vector of disease transmission among the patients they encounter and into the wider healthcare system. This sample demonstrated a relatively strong knowledge of infection prevention measures related to COVID-19. Their overall positive attitude and good infection control practices was a demonstrative effort at mitigating risks associated with the spread of the virus. The findings of this study will contribute to improving the training program to ensure that infection control practices are thoroughly embedded in daily prehospital care routines and precautions against not only COVID-19 but also general communicable diseases.

### Acknowledgments

This paper would not be possible without the support received from Hamad Medical Corporation, the Medical Research Centre team, Hamad Medical Corporation Ambulance Service management, and staff that took time to complete the survey. The authors appreciate your contribution to research.

### Conflict of interest

The authors have no conflict of interest to declare.

### Funding

No funding was received for this research.

### Ethical approval

Ethical approval for this research was received from the Medical Research Centre, with approval number MRC-05–145.

## Figures and Tables

**Table 1:** Paramedic knowledge of PPE in confirmed or suspected COVID-19 patients.

Statements	Disagree N (%)	Neutral N (%)	Agree N (%)

K1. HFNC oxygen administration to a suspected/confirmed COVID-19 patient produces aerosolized particles.	39 (13.8)	44 (15.6)	199 (70.6)

K2. A fit test for N95 masks is required to select the most appropriate mask size.	2 (0.7)	7 (2.5)	273 (96.8)

K3. Surgical masks provide paramedics adequate protection during aerosol-generating procedures for suspected/confirmed COVID-19 patients.	151 (53.6)	48 (17)	83 (29.4)

K4. ECC generates aerosolized particles of COVID-19.	14 (5.0)	26 (9.2)	242 (85.8)

K5. NIV increases the risk of aerosolized particles of COVID-19.	8 (2.8)	18 (6.4)	256 (90.8)

K6. COVID-19 particles can become aerosolized by paramedics performing aerosol-generating procedures, making airborne transmission possible.	6 (2.2)	5 (1.7)	271 (96.1)

K7. COVID-19 is predominantly transmitted by contact with contaminated surfaces or droplet transmission and touching one’s eyes, nose, or mouth.	3 (1.1)	2 (0.7)	277 (98.2)


K = Knowledge, HFNC = High flow nasal cannula, ECC = External Chest Compressions, NIV = Non-invasive ventilation.

**Table 2:** Paramedic attitudes toward PPE in confirmed or suspected COVID-19 patients.

Statements	Disagree N (%)	Neutral N (%)	Agree N (%)

A1. My employer provides me with adequate PPE to keep me safe when treating a suspected/confirmed COVID-19 patient.	11 (3.9)	14 (5)	254 (91.1)

A2. An HME filter is required when providing BVM ventilation to a suspected/confirmed COVID-19 patient.	4 (1.4)	16 (5.7)	262 (92.9)

A3. The practice of hand hygiene is required after doffing PPE to protect me from the transmission of COVID-19.	6 (2.1)	7 (2.5)	269 (95.4)

A4. Gowns should only be donned if a risk of bodily fluid cross-contamination when managing or transporting a suspected/confirmed COVID-19 patient exists.	144 (51.1)	30 (10.6)	108 (38.3)

A5. Face shields should only be donned by paramedics for aerosol-generating procedures when managing or transporting a suspected/confirmed COVID-19 patient.	143 (50.7)	44 (15.6)	95 (33.7)

A6. All paramedics should double-glove when donning PPE for the management or transport of a suspected/confirmed COVID-19 patient.	11 (3.9)	14 (5)	257 (91.1)

A7. Wearing a face shield is still required when managing a suspected/confirmed COVID-19 patient in the open environment.	8 (2.8)	19 (6.7)	255 (90.5)

A8. Doffing of PPE should be done on-scene before transporting a suspected/confirmed COVID-19 patient.	126 (44.7)	23 (8.2)	133 (47.1)

A9. Donning of PPE when managing or transporting a suspected/confirmed COVID-19 patient should be done under supervision.	49 (17.4)	60 (21.3)	173 (61.3)


A =  Attitude, PPE = personal protective equipment, HME = Heat Moisture Exchange, BVM = Bag valve mask.

**Table 3:** Paramedic practice of PPE in suspected or confirmed COVID-19 patients.

Statements	No N (%)	Yes N (%)

P1. I remove my N95 mask when performing manual ECC on a suspected/confirmed COVID-19 patient, as the procedure is exhausting.	258 (91.5)	24 (8.5)

P2. I don gloves when providing NIV to a suspected/confirmed COVID-19 patient.	29 (10.3)	253 (89.7)

P3. I avoid donning a gown in very hot weather when managing or transporting a suspected/confirmed COVID-19 patient.	245 (86.9)	37 (13.1)

P4. I re-use my N95 mask for multiple patient encounters during the same shift in the prehospital environment.	215 (76.2)	67 (23.8)

P5. When placing an ETT in a suspected/confirmed COVID-19 patient, I remove my face shield is good practice, as it mists up and obstructs my view of the vocal cords.	253 (89.7)	29 (10.3)

P6. I remove my N95 mask in the back of the ambulance when managing or transporting a suspected/confirmed COVID-19 patient.	239 (84.7)	43 (15.3)

P7. My supervisor witnesses and checks the doffing of my PPE to ensure I am following the appropriate doffing technique after managing or transporting a suspected/confirmed COVID-19 patient.	56 (19.9)	226 (80.1)

P8. I don my PPE before contacting a suspected/confirmed COVID-19 patient.	1 (0.4)	281 (99.6)


ECC = External Chest Compressions, PPE = personal protective equipment, NIV = Non-invasive ventilation, ETT = Endotracheal Tube.

**Table:** 

Code	Complete hand hygiene training at HMCAS	Tick (√)

1	< 1 year ago	

2	1 to 2 years	

3	3 to 5 years	

4	>5 years	


**Table:** 

Statement	Agree	Neutral	Disagree

A1. My employer provides me with adequate PPE to keep me safe when treating a suspected/confirmed COVID-19 patient.			

A2. An HME filter is required when providing BVM ventilation to a suspected/confirmed COVID-19 patient.			

A3. The practice of hand hygiene is required after doffing PPE to protect me from the transmission of COVID-19.			

A4. Gowns should only be donned if there exists a risk of bodily fluid cross-contamination when managing or transporting a suspected/confirmed COVID-19 patient.			

A5. Face shields should only be donned by paramedics for aerosol-generating procedures managing or transporting a suspected/confirmed COVID-19 patient.			

A6. All paramedics should double-glove when donning PPE for the management or transport of a suspected/confirmed COVID-19 patient.			

A7. Wearing a face shield is still required when managing a suspected/confirmed COVID-19 patient in the open environment.			

A8. Doffing of PPE should be done on-scene before transporting a suspected/confirmed COVID-19 patient.			

A9. Donning of PPE when managing or transporting a suspected/confirmed COVID-19 patient should be done under supervision.			


**Table:** 

Code	Gender	Tick (√)

1	Male	

2	Female	


**Table:** 

Statement	Agree	Neutral	Disagree

K1. HFNC oxygen administration to a suspected/confirmed COVID-19 patient produces aerosolized particles.			

K2. A fit test for N95 masks is required to select the most appropriate mask size.			

K3. Surgical masks provide paramedics adequate protection during aerosol-generating procedures for suspected/confirmed COVID-19 patients.			

K4. ECC generates aerosolized particles of COVID-19.			

K5. NIV increases the risk of aerosolized particles of COVID-19.			

K6. COVID-19 particles can become aerosolized by paramedics performing aerosol-generating procedures, making airborne transmission possible.			

K7. COVID-19 is predominantly transmitted through contact with contaminated surfaces or droplet transmission and touching one’s eyes, nose, or mouth.			


**Table:** 

Code	Nationality	Tick (√)

1	Tunisia	

2	Jordan	

3	Philippines	

4	South Africa	

5	United States of America	

6	United Kingdom	

7	India	

8	Other	


**Table:** 

Code	Complete N95 mask fit test at HMCAS	Tick (√)

1	< 1 year ago	

2	1 to 2 years	

3	3 to 5 years	

4	>5 years	


**Table:** 

Code	Complete HMCAS Infection Control training	Tick (√)

1	< 1 year ago	

2	1 to 2 years	

3	3 to 5 years	

4	>5 years	


**Table:** 

Code	Highest Emergency Medical Care qualification in Qatar	Tick (√)

1	Ambulance Paramedic (AP)	

2	Critical Care Paramedic (CCP)	


**Table:** 

Code	Number of years employed at HMCAS	Tick (√)

1	1 to 5	

2	6 to 10	

3	11 to 15	

4	16 to 20	

5	>20	


**Table:** 

Code	Highest level of educational	Tick (√)

1	Primary School	

2	Secondary School	

3	Level / Grade 12	

4	Diploma	

5	Bachelor’s degree	

6	Master’s degree	

7	Doctoral degree	


**Table:** 

Statement	No	Yes

P1. I remove my N95 mask when performing manual ECC on a suspected/confirmed COVID-19 patient, as the procedure is exhausting.		

P2. I don gloves when providing NIV to a suspected/confirmed COVID-19 patient.		

P3. I avoid donning a gown in very hot weather when managing or transporting a suspected/confirmed COVID-19 patient.		

P4. I re-use my N95 mask for multiple patient encounters during the same shift in the prehospital environment.		

P5. When placing an endotracheal tube in a suspected/confirmed COVID-19 patient, removing my face shield is good practice as it mists up and obstructs my view of the vocal cords.		

P6. I remove my N95 mask in the back of the ambulance when managing or transporting a suspected/confirmed COVID-19 patient.		

P7. My supervisor witnesses and checks the doffing of my PPE to ensure I am following the appropriate doffing technique after managing or transporting a suspected/confirmed COVID-19 patient.		

P8. I don my PPE before contacting a suspected/confirmed COVID-19 patient.		


## Appendix A: Online Questionnaire

### Section A: Demographic data

Instructions to participant: Please indicate your choice with a *tick (√)* in the appropriate block provided.

1. Please specify your home country *(Tick [√] one option only).*

Specify other: ____________________________________________

2. Please indicate your age in years.

______________

3. Please specify your gender *(Tick [√] one option only)* **.**

4. Please specify your highest level of education *(Tick [√] one option only)*.

5. Please specify your current designation *(Tick [√] one option only)*.

6. Please specify the number of years you have been employed as an AP or CCP at Hamad Medical Corporation Ambulance Service (HMCAS) *(Tick [√] one option only)*.

7. When did you last complete the HMCAS Infection Control training *(Tick [√] one option only)*?

8. When did you last complete an N95 mask fit test at HMCAS *(Tick [√] 1 option only)*?

9. When did you last complete the hand hygiene training at HMCAS *(Tick [√] one option only)*?

10. Based on your current knowledge of PPE use against COVID-19, please indicate your level of agreement with each of the following statements *(Tick (√) one option per statement only)*.

11. Based on your current knowledge on PPE use against COVID-19, please indicate your level of agreement with each of the following statements highlighting your attitude *(Tick [√] one option per statement only)*.

12. Based on your current practice of PPE use against COVID-19, please indicate your practice with each of the following statements *(Tick [√] one option per statement only)*.

Thank you for taking the time to complete this survey questionnaire. Please only complete this survey questionnaire once. Please ignore any further requests to complete the survey questionnaire once you have submitted your response.
